# Ultrafast Ramsey interferometry to implement cold atomic qubit gates

**DOI:** 10.1038/srep05867

**Published:** 2014-07-29

**Authors:** Jongseok Lim, Han-gyeol Lee, Sangkyung Lee, Chang-Yong Park, Jaewook Ahn

**Affiliations:** 1 Department of Physics, KAIST, Daejeon 305-701, Korea; 2 Korea Research Institute of Standards and Science, Daejeon 305-340, Korea; 3 Current address: Centre for Cold Matter, Blackett Laboratory, Imperial College London, Prince Consort Road, London SW7 2AZ, United Kingdom.

## Abstract

Quantum computing is based on unitary operations in a two-level quantum system, a qubit, as the fundamental building block, and the ability to perform qubit operations in an amount of time that is considerably shorter than the coherence time is an essential requirement for quantum computation. Here, we present an experimental demonstration of arbitrary single-qubit *SU(2)* quantum gate operations achieved at a terahertz clock speed. Implemented by coherent control methods of tailored ultrafast laser interaction with cold rubidium atomic qubits, Bloch vector manipulation about all three rotational axes was successfully demonstrated. The dynamic evolution of the qubits was successfully measured by devised femtosecond Ramsey interferometry. We anticipate this demonstration to be a starting point to process quantum algorithm in a simplified manner by a programmed sequence of femtosecond laser pulses.

The popularization of computers has significantly influenced many aspects of human life and also unprecedentedly elevated production efficiency in industry^1^. To meet the need for faster computations, significant efforts have been invested by industry and academia^2^. Now the clock speed of a CPU has reached 10 GHz, and in an attempt to further improve computational speeds, new types of computer architectures are under active investigation^3^,^4^,^5^. Quantum computing, in particular, has drawn much interest because of its enhanced capabilities based on quantum mechanics, and quantum computers are expected to out-perform classical computers^6^,^7^,^8^.

Prototype quantum computers have been developed over the past decades in various quantum systems including trapped ions^9^, nuclear spins of molecules^10^, electron spins of quantum dots^11^,^12^,^13^, Rydberg atoms^14^,^15^, and superconductor junctions^16^. Areas where quantum computation appears to have a significant advantage are in factoring numbers and in searching databases^6^, both of which use the massive Hilbert space of a large number of entangled qubits. Dealing with many qubits within the time limited by the coherence has been a challenging task, so techniques for doing this with high speed could be of great interest.

The first requirement of quantum computing is the ability to control the state of single qubits. Arbitrary *SU(2)* logical gates of single qubits can be constructed with qubit rotations about at least two distinct axes^7^,^8^. Here, we experimentally demonstrate ultrafast quantum gate operations achieved on a femtosecond time scale. Coherent control methods^17^,^18^,^19^,^20^,^21^ that utilize tailored short optical pulses are used to manipulate a qubit composed of electronic states of atomic rubidium (Rb). Rabi rotations^22^,^23^,^24^, including the *z*-rotation, are implemented by tailored laser pulses, and the phase evolution dynamics were measured using an ultrafast version of Ramsey interferometry^25^ devised with a phase-coded laser pulse sequence^26^,^27^,^28^. Finally, the feasibility of sequential gate operations was demonstrated with programmed Rabi rotations achieved with six programmed femtosecond pulses at terahertz computational speeds.

The qubit |ψ(t) > = *α*_0_(*t*)|0 > + *α*_1_(*t*)|1 >, is modeled as the two lowest electronic states of Rb, the ground state |0 > = |5S_1/2_ > and the excited state |1 > = |5P_1/2_ >. When the qubit interacts with a frequency-resonant pulse, for which the electric field is given by 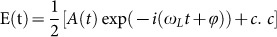 where *A*(*t*) is the Gaussian envelope, *ω_L_* is the center frequency (resonant to the qubit angular frequency *ω_o_*), and *φ* is the phase of the laser pulse, the time-evolution operator, or the quantum operator, in the strong-field interaction regime^29^,^30^,^31^ is then given by 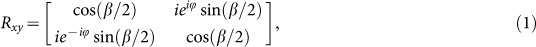
where 
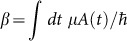
 is the pulse area of the laser pulse, and *μ* is the transition dipole moment for the |0 >→ |1 > transition (see section ‘Quantum Operators' in the Supplementary Information). Equation (1) is the rotation operation about an axis 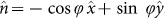. Therefore we can implement the *x*- and *y*-rotation operators among many others by frequency-resonant pulses with phase *φ* = *π* and *φ* = *π*/2, respectively, and the rotation angle is defined by *β*. Also, we can implement the rotation about the 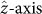, identical to the phase-shift gate, based on the quantum nature of energy-level shifting by light. When a far-detuned strong laser pulse, i.e., 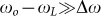 and Δ*ω* is the bandwidth of the pulse, induces a dispersive interaction, the two energy levels of the qubit bend toward opposite directions from each other, thereby resulting in the so-called dynamic Stark shift^32^ (DSS), and the instantaneous energy shift causes additional phase evolution of the qubit to the natural frequency of the phase evolution, *ω_o_*. The quantum operator induced by the far-detuned pulse interaction gives the phase-shift gate as 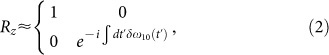
where *δω*_10_(*t*) = *δω*_1_(*t*) − *δω*_0_(*t*), and 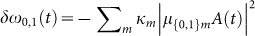 are the time-varying angular frequencies of the DSS of |0 > and |1 >, respectively, with the phase-shift 
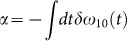
. For an experimental demonstration of the ultrafast optical qubit manipulations, we used the experimental setup depicted in Fig. 1 (see experimental details in Methods). In particular, uniform-intensity laser-atom interactions are achieved by spatial confinement of Rb atoms tightly trapped in a magneto-optical trap^33^,^34^ (MOT).

The first qubit manipulation experiment in Fig. 2 aims the direction control of the rotation axis 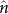 in the *x-y* plane that was performed with the two Ramsey pulses (R1 and R2) probed by the ionization pulse of which the arrival time was swept in the time window of [−0.5 ps, 2.5 ps] to monitor the change in the |1 > state probability. The two Ramsey pulses were divided from a single laser pulse by a programmed acousto-optic pulse-shaper^35^,^36^ (AOPS) to have the programmed phase difference Δ*φ* and the fixed pulse area and delay of *β* = π/2 and τ = 1.5 ps, respectively. Figure 2a shows the transient evolution of the |1 > state probability (circles for the experiments and lines for the theoretical calculations) according to the phase difference. The Bloch vector along with the 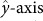, initially moved from the north pole by the first pulse as shown in the inset of Fig. 2a, was rotated about the controlled rotation axis 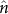 by the second pulse. When Δ*φ* was varied from zero to *π*, the rotation axis was continuously changed from 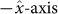 to 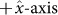 as illustrated in Fig. 2 for (b) Δ*φ* = 0, (c) π/4, (d) π/2, (e) 3π/4, and (f) π.

The second experiment demonstrates the phase-shift gate operation performed by the far-detuned pulse and measured with the ultrafast Ramsey interferometry (see Methods for details). After the interaction with a far-detuned short pulse (the phase-shift pulse) sandwiched between R1 and R2, the probability of the |1 > state is given by 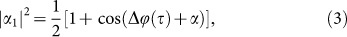
where the phase difference Δφ was coded as a function of the time delay τ by Δφ = C × τ, with a fixed constant C/2π = 1 THz. The measured probability of |1 > is color-mapped in Fig. 3a as a function of τ and *β_Z_*, the pulse area of the phase-shift pulse. The probability exhibits cosine-like oscillations, as predicted by Eq. (3), and the second oscillation peaks after the phase-shift pulse (t = 730 fs) drifted from approximately 1000 fs to later times as *β_Z_* increases, in agreement with the theoretical calculation indicated by the dashed gray line. Figure 3b shows the measured the phase-shift *α* as a function of *β_Z_*, extracted via a numerical fit of the Ramsey fringes in the time region of [750 fs, 1750 fs] to Eq. (3). Figure 3c shows the experimental Ramsey fringe data (circles) for *α* = 0 (blue), *α* = *π* (red), and *α* = 2*π* (gray), where *α* is the angle of the 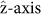 rotation, compared with the calculations (dashed lines) of the time-dependent solution of the Schrödinger equation. When spatial intensity averaging effect^37^ is taken into account, a portion of the Rb atoms acquires a smaller phase shift, which results in the decreased contrast of the Ramsey fringe (solid lines). The time evolution of the Bloch vector, which is shown in Fig. 3e, is successfully reconstructed for *α* = *π* using the phase and amplitude measurements in Fig. 3c and Fig. 3d, respectively. The coherent transient effect^38^,^39^ causes the oscillatory behavior around the equator of the Bloch sphere and the Bloch vector successfully ends along the –*y*-direction after the phase-shift pulse. The measured oscillation amplitude (circles) is smaller than the calculation (solid line) due to the limited time resolution of the ionization pulse of about 150 fs in this specific part of the experiment.

Finally, to verify the feasibility of sequential gate operations, we employ the femtosecond pulse-tailoring technique further to produce sequences of six *π*/2-pulses with controlled phase combinations (see Methods for details). When the six pulses have a phase combination of *φ* = [0, *π*, 0, 0, *π*, 0], the time-fragmented change in probability for |1 > is given by [0 → 0.5 → 0 → 0.5 → 1 → 0.5 → 1]. Figure 4 shows the experimental results in red circles compared with the Schrödinger equation calculation, which is shown as a red line. When *φ* = [0, 0, *π*, *π*, *π*, 0], the probability change (red circles and line) is [0 → 0.5 → 1 → 0.5 → 0 → 0.5 → 0]. This demonstration of time-fragmented Rabi rotations confirms that ultrafast quantum gates can operate at a speed of 1 THz.

Qubits in quantum computation are the fundamental passive elements of the machine. There are advantages of using atomic qubits in quantum computing, not the least of which is ease of implementation, but individual neutral atoms can be essentially immune from decoherence, which is a major problem faced by other implementation of quantum algorithms. Recent achievements of the atom entanglement based on the Rydberg atom dipole blockade effect^40^ and the atom arrangement in a designer's optical lattice^41^ hold promise for neutral atom quantum computing.

In conclusion, we have demonstrated the optical implementation of ultrafast quantum gates of atomic qubits. We devised a Ramsey-type temporal interferometric measurement of single qubits to confirm the coherent control of all *SU(2)* qubit rotations. The demonstrated scheme has performed single-qubit gates at an operational clock speed as high as 1 THz. The improvement of the operating speed benefits the computational power of a quantum computer by enabling a huge number of operations within a limited coherence time. Furthermore, the coherent control method could simplify otherwise complex optical implementation of a quantum circuit, replacing heterogeneous optical control sources by a temporally- and spectrally-programmed pulse sequence from a single ultrafast laser source.

## Methods

### Experimental Setup

**Figure 1:**
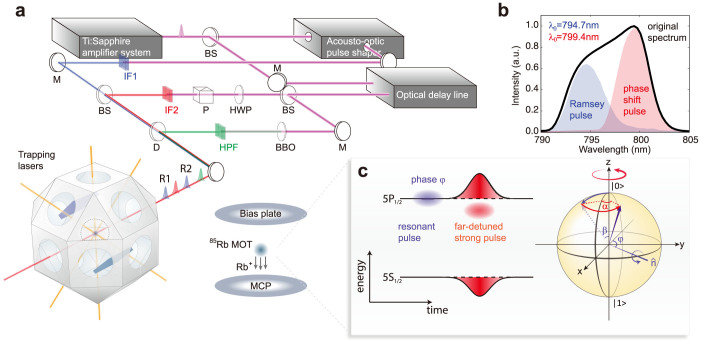
Schematic of the experimental setup. (a), The femtosecond pulses are divided into three beam lines: (A) the resonant Ramsey pulses R1 and R2, (B) the phase-shift pulse, and (C) the ionization pulse. The three beam lines are guided to Rb atoms trapped in the MOT chamber. (M, mirror; HWP, half-wave plate; P, polarizer; BBO, barium borate plate; IF1, interference filter at 794.7 nm; IF2, interference filter at 799.7 nm; HPF, high-pass filter at 500 nm; BS, beamsplitter; D, dichroic mirror.) (b), The spectra of the laser pulses are indicated in blue for the resonant pulses and in red for the phase-shift pulse, compared with the initial pulse spectrum in a solid black line. (c), The energy level change induced by the phase-shift pulse and the corresponding *z*-rotation of the Bloch vector.

We used a Ti:sapphire laser amplifier system generating 200 fs pulses at a repetition rate of 1 kHz with pulse energy of 1.0 mJ which were delivered in a beam of 3 mm diameter. The spectrum of the laser, shown in Fig. 1b as a black solid line, was shaped to efficiently cover the spectra of both the resonant Ramsey pulse (λ*_L_* = 794.7 nm with a bandwidth of 4 nm) and the far-detuned pulse (λ*_L_* = 799.7 nm with a bandwidth of 3 nm). The ionization pulse was frequency-doubled in a BBO crystal and filtered by a high frequency pass filter, and the spectrum was centered at 399 nm with a bandwidth of 3 nm. The atoms (^85^Rb) were cooled and trapped in a standard MOT, with the density of 10^9^/cm^3^ and the temperature of hundreds of *μ*K. The trapping and repumping lasers of the MOT were blocked by a mechanical shutter at t = −200 *μ*s to ensure Rb atoms initially in the ground state. The shutter was reopened after t = 3 ms, and operated at 2 Hz to reboot the MOT. Another shutter operated at the same frequency was used in the femtosecond beam line. The overall experimental procedure is illustrated in Supplementary Figure S1.

The beam line A had two resonant pulses with a time delay τ, which rotated the Bloch vector to an arbitrary point in the Bloch sphere. The first and second pulses (R1 and R2) rotated the Bloch vector about 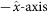 and the rotation axis 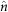, respectively, where Δ*φ* = *φ*_2_ − *φ*_1_ is the phase difference between the pulses. The phase difference Δ*φ* and the time delay τ were tailored by the AOPS. Along the beam line B, a far-detuned pulse performed the *z*-rotation, which was induced by the DSS as schematically illustrated in Fig. 1c. Then, the frequency-doubled pulse in the beam line C measured the |1 > state population of the qubit by ensemble measurement through photo-ionizing. The evolution of the |1 > state population was obtained as a function of time with the resolution of 200 fs by changing the time delay between the quantum gate pulses and the ionization pulse with a translation stage. The produced ions in the MOT were accelerated toward a micro-channel plate detector (MCP) by an electric field of 70 V/cm, and the number of ions was measured by the MCP. Then, the ion signal was recorded by a boxcar signal processor. The effective detection efficiency was about 10%, and the probability of |1 > was normalized by the Rabi oscillation measurement at the pulse area of *π* illustrated in Supplementary Figure S2.

The number of atoms in the interaction region was 8 × 10^3^. The spatial intensity averaging effect was minimized with the diameter of the Rb MOT adjusted to 250 *μ*m by controlling the beam sizes of the trapping and repumping lasers of the MOT. The resonant pulses performed the *x*- and *y*-rotations, and the far-detuned pulse performed the *z*-rotation, respectively, of the qubit. The femtosecond laser beams were spatially overlapped by a beam splitter and a dichroic mirror, and slowly focused onto the Rb cloud in the MOT with a 500 mm focal length lens. The diameter of the focused beam spot was chosen to be 700 *μ*m through adjusting the distance between the lens and the MOT to reduce the spatial averaging effect.

### Ultrafast Ramsey interferometry

For the experimental retrieval of the qubit phase evolution during the phase-shift gate operation, an ultrafast Ramsey interferometry is devised. In this scheme, the phase difference of the second Ramsey pulse (R2) relative to the first Ramsey pulse (R1) is coded as a function of the time delay τ by Δφ = C × τ, with a fixed constant C (in our experiments, C/2π = 1 THz). Then, when the qubit initially in the ground state |0 > undergoes sequential operations of the far-detuned pulse (the phase-shift pulse) sandwiched by the two Ramsey pulses with the fixed pulse area of *π*/2, the qubit manipulation is given by 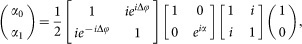
and the probability of |1 > is obtained as in Eq. (3). The final |1 > state probability |*α*_1_|^2^ gives a cosine-like oscillation as a function of τ with the period of 1/C, and the phase shift *α* appears in the shifted Ramsey oscillation phase. Therefore, by comparing the shifted Ramsey oscillation to the original Ramsey fringe (*α* = 0 case), the amount of the phase-shift gate operation is extracted as well as the phase evolution during the interaction.

For the phase-shift gate experiment, we used total four laser pulses; two Ramsey pulses, one far-detuned pulse, and one ionization pulse. The AOPS controlled both the time delay and the phase difference between the two Ramsey pulses. The time of R1 was fixed at zero, while the time of R2 was varied from −400 fs to 2100 fs. The time of the phase-shift pulse, or the far-detuned pulse, was fixed at 730 fs. The energy of the pulse was varied from zero to 12 *μ*J which corresponded to the pulse area *β_Z_* = 4.7π by rotating a half-wave plate followed by a fixed polarizer. The time of the ionization pulse was also fixed at 10 ps for the measurement of the final probability of |1 >, and the Ramsey signal was recorded as a function of τ.

### Programmed Rabi rotations

For this experiment, we used six resonant pulses which had pulse areas of *π*/2 and controlled phase combinations. First, three pulses, separated by 1 *ps* each, were produced from the AOPS, and then the number of pulses was doubled by a 50/50 optical beam splitter. By introducing optical path-length difference between the two beam lines that corresponded to *τ_c_* = 4 ps, six pulses of fixed time delays were produced, and sequentially applied to the qubits. The additional phase of the copied pulses *φ_c_* was controlled by the fine tuning of the length of the split arms as *φ_c_* = −*ω_L_* × *τ_c_*. We used two phase combinations of the first three pulses, *φ* = [0, *π*, 0] and *φ* = [0, 0, *π*], and *φ_c_* was adjusted in each combination to make *φ* = [0, *π*, 0, 0, *π*, 0] (*φ_c_* = 0) and *φ* = [0, 0, *π*, *π*, *π*, 0] (*φ_c_* = *π*), respectively. The probability change was measured as a function of time by varying the arrival time of the ionization pulse in the time window of [−3 ps, 8.5 ps].

## Supplementary Material

Supplementary Information Supplementary information for Ultrafast Ramsey interferomentry to implement cold atomic qubit gates

## Figures and Tables

**Figure 2:**
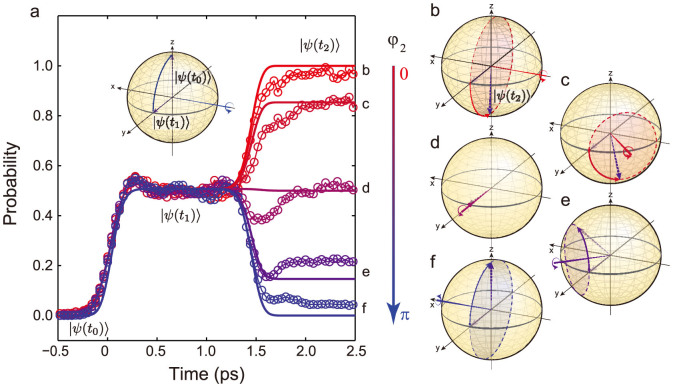
*x*- and *y*-rotations of the Bloch vector. (a), The probability of |1 > versus time. The two *π*/2 Rabi rotations arrived at time zero and 1.5 ps, respectively, and the phase difference was varied from zero to π as Δ*φ* = *nπ*/4 (*n* = 0…4). (b–f), The time evolutions of the qubit are correspondingly illustrated in Bloch spheres.

**Figure 3:**
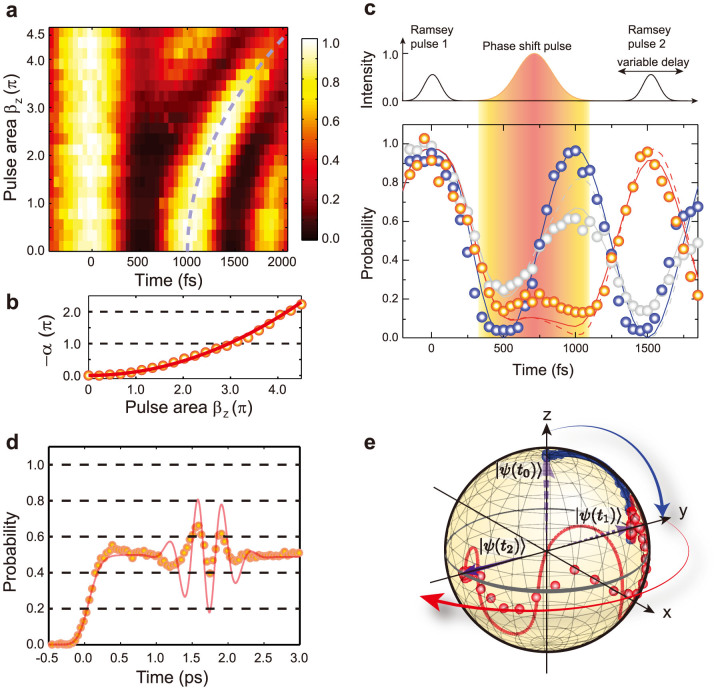
Experimental demonstration of phase shift gates. (a), The probability of |1 > as a function of the delay between two Ramsey pulses and the pulse area (*β_Z_*) of the phase-shift pulse. (b), The measured phase shift versus the pulse area. Circles show the values extracted from the numerical fit to Eq. (3). The solid line is a theoretical calculation given as the square of the pulse area. (c), Ramsey fringes for α = 0 (blue), α = π (red), and α = 2π (gray). Experimental results are shown as circles, and calculations that consider the spatial averaging effect are given as solid lines. The calculations without the spatial averaging effect are plotted in dashed lines. The shaded region in the figure emphasizes the interaction time interval of the phase-shift pulse. (d), The probability evolution induced by the phase-shift pulse was measured by photo-ionizing the |1 > state. The qubit interacted first with the *π*/2-pulse (R1) at t = 0 and then with the phase-shift pulse for *α* = *π* at t = 1.7 ps. The ionization pulse was swept in the time window of [−1 ps, 3 ps]. The measured probability of |1 > (circles) is compared with the calculation (solid line). (e), The time evolution of the Bloch vector interacting with R1 and the phase-shift pulse for *α* = *π*.

**Figure 4:**
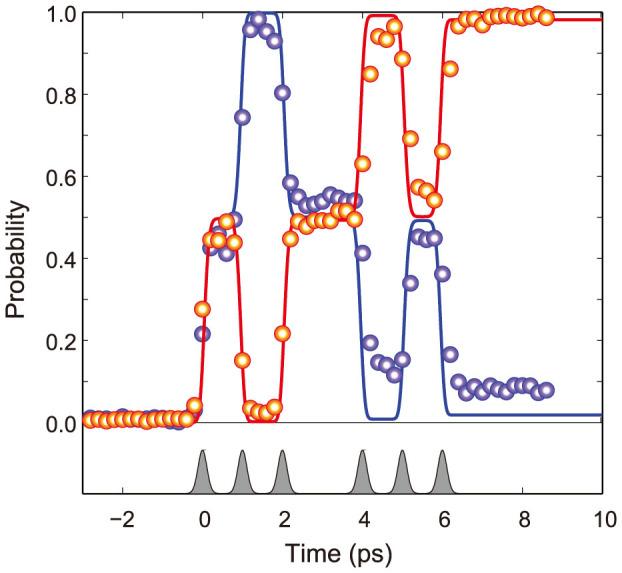
Programmed Rabi rotations. The |1 > state population of the qubit versus time. The two sequences of six *π*/2-pulses have phase combinations *φ* = [0, *π*, 0, 0, *π*, 0] (red) and *φ* = [0, 0, *π*, *π*, *π*, 0] (blue), respectively.
