# Supplementary material for: Ultrafast Ramsey interferometry to implement cold atomic qubit gates
Source: Sci Rep. 2014 Jul 29;4:5867. doi: 10.1038/srep05867 (PMC5376194; doi:10.1038/srep05867)
Supplement: Supplementary Information — for Ultrafast Ramsey interferomentry to implement cold atomic qubit gates [file srep05867-s1.pdf]

Supplementary Information

Ultrafast Ramsey interferometry to implement cold atomic qubit gates

Jongseok Lim[1], Han-gyeol Lee[1], Sangkyung Lee[2], Chang Yong Park[2], and Jaewook Ahn[1*]
[1]Department of Physics, KAIST, Daejeon 305-701, Korea
[2]Korea Research Institute of Science and Standards, Daejeon 305-340, Korea
*Correspondence to: jwahn@kaist.ac.kr

Quantum Operators

The Hamiltonian of the atomic qubit interacting with the laser pulse is given by

$$H = \begin{bmatrix} \hbar\omega_g & 0 \\ 0 & \hbar\omega_e \end{bmatrix} + \begin{bmatrix} 0 & -\mu\cdot E(t) \\ -\mu\cdot E(t) & 0 \end{bmatrix}, \qquad \text{(S1)}$$

where $\hbar\omega_i$ is the energy of the state $|\mathrm{i}>$, μ is the dipole moment, and $E(t)$ is the laser E-field. In the interaction picture, the time-dependent Schrödinger equation is given by

$$\begin{bmatrix} \dot{a}_0(t) \\ \dot{a}_1(t) \end{bmatrix} = \begin{bmatrix} 0 & i\frac{\Omega(t)}{2}e^{i\varphi} \\ i\frac{\Omega(t)}{2}e^{-i\varphi} & 0 \end{bmatrix} \begin{bmatrix} a_0(t) \\ a_1(t) \end{bmatrix}, \qquad \text{(S2)}$$

where $\Omega(t) = \mu A(t)/\hbar$ is the Rabi frequency and the rotating-wave approximation is used ignoring the Bloch-Siegert shift. When $\hbar\omega_g$ is taken to be zero and the laser satisfies the resonant condition, $\omega_L = \omega_0$, the interaction picture Hamiltonians at different times commute. So the time evolution operator after the end of the pulse is given by

$$R_{xy} = \exp\begin{bmatrix} 0 & \int_{-\infty}^{\infty} \mathrm{dt}' \frac{\Omega(t')}{2} e^{i\varphi} \\ \int_{-\infty}^{\infty} \mathrm{dt}' \frac{\Omega(t')}{2} e^{-i\varphi} & 0 \end{bmatrix}$$

$$= \begin{bmatrix} \cos(\beta/2) & ie^{i\varphi}\sin(\beta/2) \\ ie^{-i\varphi}\sin(\beta/2) & \cos(\beta/2) \end{bmatrix}, \qquad \text{(S3)}$$

where $\beta = \int dt' \, \Omega(t')$ is the pulse area of the laser pulse. Equation (S3) is the rotation operation about a vector $\hat{n}$ given by $\hat{n} = -\cos\varphi\,\hat{x} + \sin\varphi\hat{y}$. The electric field of the two pulses, time-separated by τ, that are generated by the pulse-shaper, is given by

$$\mathrm{E(t)} = A(t)\cos(\omega_L t + \varphi_{CEP}) + A(t-\tau)\cos(\omega_L t + \varphi_{CEP} + \Delta\varphi). \tag{S4}$$

Note that we take the carrier-envelope phase of the initial pulse, φ_{CEP} in Eq. (S4), to be zero since the overall phase of each pulse does not affect the experiment. Additionally, in our experimental condition, φ_{CEP} little affects the amplitude of the laser pulse since there are about one hundred of oscillations inside the envelope. $\Delta\varphi$, the phase difference between the pulses, is changed by the pulse-shaper and controls the axis of rotation of the Bloch vector.

When the laser pulse is detuned from the resonance frequency of the qubit, $\omega_L \neq \omega_0$, the axis of rotation strays away from the equator. The matrix differential equation in Eq. (S2) predicts the evolution of the qubit as following:

$$\begin{bmatrix} a_0(t) \\ a_1(t) \end{bmatrix} \cong \begin{bmatrix} \cos(\beta'/2) + i\frac{\Delta_0}{\Omega'}\sin(\beta'/2) & i\frac{\Omega}{\Omega'}\sin(\beta'/2) \\ i\frac{\Omega}{\Omega'}\sin(\beta'/2) & \cos(\beta'/2) - i\frac{\Delta_0}{\Omega'}\sin(\beta'/2) \end{bmatrix} \begin{bmatrix} a_0(0) \\ a_1(0) \end{bmatrix}, \tag{S5}$$

where $\beta'(t) = \int_{-\infty}^{t} dt' \, \Omega'(\mathrm{t'})$ is the generalized pulse area at time t, $\Omega'(\mathrm{t}) = \sqrt{\Omega^2(t) + {\Delta_0}^2}$ is the generalized Rabi frequency, and $\Delta_0 = \omega_L - \omega_0$ is the detuning. Note that the direction of the rotation axis varies during the interaction since the generalized Rabi frequency changes with the envelope of the pulse $A(t)$.

Further increasing of the detuning, *i.e.,* $\omega_o - \omega_L \gg \Delta\omega$, gives the time evolution of the qubit as

$$\begin{bmatrix} a_0(t) \\ a_1(t) \end{bmatrix} \cong \begin{bmatrix} e^{i\alpha_0(t)} & \frac{\Omega(t)}{\Delta_0} e^{i\Delta_0 t} \\ \frac{\Omega(t)}{\Delta_0} e^{i\Delta_0 t} & e^{i\alpha_1(t)} \end{bmatrix} \begin{bmatrix} a_0(0) \\ a_1(0) \end{bmatrix}, \tag{S6}$$

where $\alpha_{0,1}(t) = -\int_{-\infty}^{t} dt'\ \delta\omega_{0,1}(t)$, $\delta\omega_i = -\sum_m \frac{|\mu_{im}A(t)|^2}{2\hbar^2}\frac{\omega_{mi}}{\omega_{mi}^2-\omega_L^2}$ is the dynamic Stark shift (DSS) of $|i>$ coupled with the state $|\mathrm{m}>$, μ_{im} is the transition dipole moment for the $|\mathrm{i}>\rightarrow|\mathrm{m}>$ transition, and $\omega_{ij} = \omega_i - \omega_j$. The calculation considers $|5\mathrm{S}_{1/2}>$, $|5\mathrm{P}_{1/2}>$, and $|5\mathrm{P}_{3/2}>$ for the state $|\mathrm{m}>$. Then, the Bloch vector oscillates in time around the equator with the oscillation frequency of Δ_0, and the oscillation amplitude is given proportional to $\frac{\Omega(t)}{\Delta_0}$. When Eq. (S1) is rearranged as

$$H = \begin{bmatrix} \hbar\delta\omega_0 & 0 \\ 0 & \hbar(\omega_1+\delta\omega_0) \end{bmatrix} + \begin{bmatrix} -\hbar\delta\omega_0 & -\mu\cdot E(t) \\ -\mu\cdot E(t) & -\hbar\delta\omega_0 \end{bmatrix}, \tag{S7}$$

the interaction picture description allows zero ground state energy in the presence of the DSS. Then, the time evolution of the qubit after the pulse is given by

$$\begin{bmatrix} a_0 \\ a_1 \end{bmatrix} = \begin{bmatrix} 1 & 0 \\ 0 & e^{i\alpha} \end{bmatrix} \begin{bmatrix} a_0(0) \\ a_1(0) \end{bmatrix}, \tag{S8}$$

which results in the phase-shift gate in Eq. (2).

Rabi oscillation

Supplementary Figure S2 shows the experimental results (circles) of the Rabi oscillation compared with the calculation (dashed line) as a function of the calculated pulse area. The pulse area was varied by the programmable acousto-optic pulse-shaper. Note that, due to the Gaussian spatial intensity profile of the laser pulses, the oscillation amplitude of the experimental results was measured smaller than the simple calculation (dashed line). When the spatial averaging effect was taken into account, the calculation (solid line) better fits the experimental results. In the experiment, the probability of $|1>$ was calibrated by the Rabi oscillation measurement at the pulse area of π.

Programmed Rabi rotations

For the experiment in Fig. 4, we used six resonant pulses which had respectively pulse areas of $\pi/2$ and controlled phase combinations. From Eq. (S3), the quantum operation of a $\pi/2$-pulse with a phase $\varphi = 0$ is given by

$$\left(\frac{\pi}{2}\right)_0 = \frac{1}{\sqrt{2}}\begin{bmatrix} 1 & i \\ i & 1 \end{bmatrix}. \quad \text{(S9)}$$

And the quantum operation of a $\pi/2$-pulse with a phase $\varphi = \pi$ is given by

$$\left(\frac{\pi}{2}\right)_\pi = \frac{1}{\sqrt{2}}\begin{bmatrix} 1 & -i \\ -i & 1 \end{bmatrix}, \quad \text{(S10)}$$

which is the inverse matrix of Eq. (S9). Therefore, if we sequentially apply the two $\pi/2$-pulses with a phase combination of $\varphi = [0, \pi]$, then the $\left(\frac{\pi}{2}\right)_\pi$ pulse brings back the state of the qubit changed by the $\left(\frac{\pi}{2}\right)_0$ pulse. So, the $\left(\frac{\pi}{2}\right)_\pi$ pulse is the “inverse gate” of the $\left(\frac{\pi}{2}\right)_0$ pulse. First, three pulses were generated from the acousto-optic pulse-shaper (AOPS), and then the number of pulses was doubled by a 50/50 beam splitter. By introducing optical path-length difference between the two beam lines that corresponded to τ_c= 4 ps, the six pulses of fixed time delays were produced. These pulses were then sequentially applied to the ensemble of qubits. Then, the net electric field of the six pulses is given by

$$\begin{aligned} \mathrm{E(t)} = {} & A_1(t)\cos(\omega_L t) + A_2(t)\cos(\omega_L t + \varphi_2) + A_3(t)\cos(\omega_L t + \varphi_3) + \\ & A_4(t)\cos(\omega_L t + \varphi_c) + A_5(t)\cos(\omega_L t + \varphi_2 + \varphi_c) + A_6(t)\cos(\omega_L t + \varphi_3 + \varphi_c), \end{aligned} \quad \text{(S11)}$$

where $A_i(t)$ and φ_i are the envelope and the relative phase of the *i*-th pulse, respectively, and φ_c is the phase offset resulted from the split arms given by $\varphi_c = -\omega_L \times \tau_c$. We used two phase combinations of the first three pulses, $\varphi = [0, \pi, 0]$ and $\varphi = [0, 0, \pi]$, and φ_c was adjusted in each combination to make $\varphi = [0, \pi, 0, 0, \pi, 0]$ $(\varphi_c = 0)$ and $\varphi = [0, 0, \pi, \pi, \pi, 0]$ $(\varphi_c = \pi)$, respectively. The probability change was measured as a function of time by varying the time of the ionization pulse in the time window of [-3 ps, 8.5 ps]. Note

that φ_c was drifted during the experiments due to the unwanted fluctuation of the path length difference of the split arms, but the phase fluctuation was slow enough relative to the time for one experiment cycle (about one minute). The length of each split arm was 15 cm. The presented data was post-selected based on the five data points at around t=8 ps.

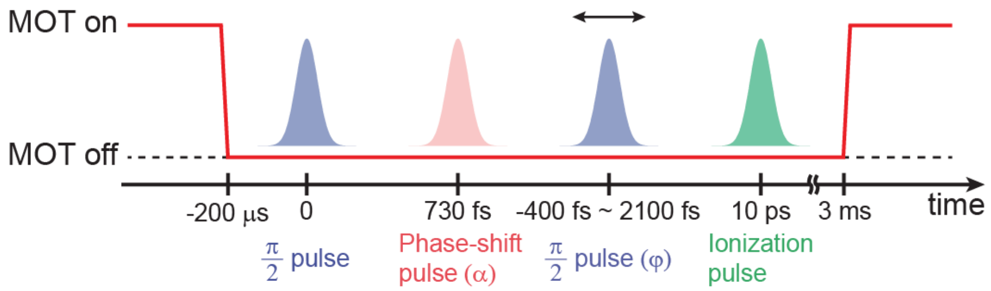

Supplementary Figure S1. Operation time sequence of laser pulses and MOT. The four laser pulses in Ramsey interferometry; two Ramsey pulses (blue), the phase-shift pulse (red), and the ionization pulse (green). The lasers for the MOT were blocked at -200 μs before the Ramsey pulses and reopened after 3 ms. The overall operational cycle was 2 Hz.

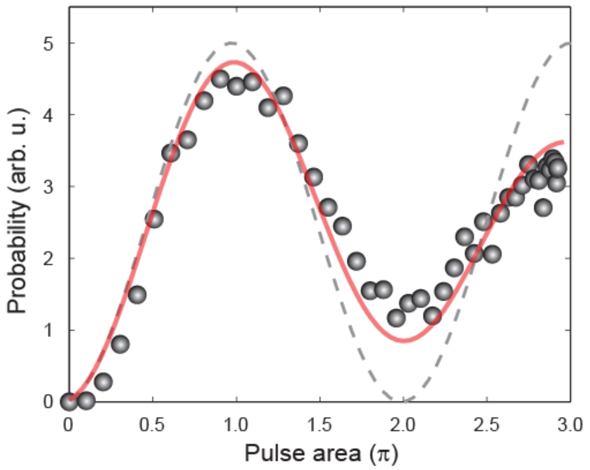

Supplementary Figure S2. Rabi oscillation. Experimental results (circles) of the probability of $|\mathbf{1}>$ compared with the numerical calculation (dashed line) as a function of pulse area. The solid line (red) shows another calculation considering the spatial averaging effect.
